# Recombination Shapes Genome Architecture in an Organism from the Archaeal Domain

**DOI:** 10.1093/gbe/evu003

**Published:** 2014-01-03

**Authors:** David J. Krause, Xavier Didelot, Hinsby Cadillo-Quiroz, Rachel J. Whitaker

**Affiliations:** ^1^Department of Microbiology, University of Illinois at Urbana-Champaign; ^2^Department of Infectious Disease Epidemiology, Imperial College London, London, United Kingdom; ^3^School of Life Sciences, Arizona State University

**Keywords:** Archaea, recombination, *Sulfolobus islandicus*

## Abstract

Variation in recombination rates across chromosomes has been shown to be a primary force shaping the architecture of genome divergence. In archaea, little is known about variation in recombination across the chromosome or how it shapes genome evolution. We identified significant variations in polymorphism occurring across the chromosomes of ten closely related sympatric strains of the thermoacidophilic archaeon *Sulfolobus islandicus*. Statistical analyses show that recombination varies across the genome and interacts with selection to define large genomic regions with reduced polymorphism, particularly in the regions surrounding the three origins of replication. Our findings demonstrate how recombination defines the mosaic of variation in this asexually reproducing microorganism and provide insight into the evolutionary origins of genome architecture in this organism from the Archaeal domain.

## Introduction

Spurred by the rapid increase in genome sequencing, there is a growing interest in the genomic architecture of divergence, which integrates evolutionarily driven genome dynamics with chromosomal organization (Feder et al. 2005; Roselius et al. 2005; Stevison et al. 2011; Nosil and Feder 2012; Via 2012). In current evolutionary models for sexual eukaryotes, the relationship between mutation, selection, and recombination as each of these processes varies across the chromosome explains the architecture of divergence. For example, recent genomic studies have shown that reduced recombination in proximity to centromeres (Choo 1998) or large chromosomal inversions (Stevison et al. 2011) as well as increased recombination at recombination hotspots (Myers et al. 2008) can greatly influence the way diversity is distributed across the genome. This is because through recombination–selection antagonism, physical linkage between loci defines the extent to which diversity in the genome is purged by natural selection, whether by background selection against deleterious genotypes or genetic hitchhiking of adaptive alleles (Felsenstein 1974). A better understanding of these forces and how their interactions shape diversity promises to reveal the genetic basis of phenotypic variation and the evolutionary processes of natural selection, adaptation, divergence, and speciation (Nosil and Feder 2012; Via 2012).

Bacteria and archaea reproduce asexually; therefore recombination is not an obligate part of each generation. Therefore, it has long been assumed that there is little room for recombination–selection balance in genome dynamics of organisms from these domains. However, through comparative genomics of closely related bacteria, the influence of recombination has been suggested to vary across the chromosome, suggesting that these evolutionary mechanisms may exist in bacterial genomes (Touchon et al. 2009). Little is known about how recombination affects the genome evolution of organisms from the Archaeal domain of life. In archaea, proteins involved in replication, recombination, and repair are more closely related to their homologs in the sister domain eukaryotes than to those in bacteria, suggesting that these cellular processes may differ dramatically from organisms in the bacterial domain (Woese and Fox 1977; Cann and Ishino 1999). Multi-locus sequence analysis of natural archaeal populations has shown that in both the crenarchaeal and the euryarchaeal divisions, recombination can occur at rates higher than mutation (Papke et al. 2004; Whitaker et al. 2005), and these levels also fall within the range associated with bacterial recombination (Vos and Didelot 2009). Genome analysis of 12 *S. islandicus* strains isolated from a single hot spring shows that recombination occurs more frequently within than between species (Cadillo-Quiroz et al. 2012), demonstrating the influence of gene flow on diversity in this system.

In the laboratory, it has been demonstrated that in *Haloferax*, a member of the Euryarchaea, cell–cell fusions result in recombinant transfer of donor DNA in one long (>300 kb) tract but suggest the potential for recombination to occur throughout the chromosome with consequences similar to crossing over in eukaryotes (Cohan and Aracena 2012; Naor et al. 2012).

In *Sulfolobus*, the model organism of the Crenarchaea, transfer of plasmids among *Sulfolobus* strains has been observed (Schleper et al. 1995), and genetic transfer of chromosomal markers independent of plasmid-mediated conjugation appears to occur through a conjugation-like mechanism (Ajon et al. 2011). Recombination readily occurs between mutations that are as few as 10 bp apart (Hansen et al. 2005), and it has been demonstrated to involve a mechanism that resembles lambda red requiring as little as 2–5 bp homology on one side of a mutation (Grogan and Stengel 2008). Incorporation of DNA acquired through in vitro transformation has been shown to involve discontinuous short tracts (Grogan and Rockwood 2010). Tracking of multiple unselected markers in recombinant clones suggested that recombination occurs throughout the chromosome (Grogan 1996). Despite experimental and molecular evidence that recombination occurs, whether or how recombination might vary across the archaeal chromosome is not known. Without this piece of the evolutionary puzzle, it is impossible to accurately determine how genome diversity arises and is maintained.

*Sulfolobus islandicus* is a thermophilic crenarchaeon that is readily cultured from acidic hot springs. Like other members of the *Sulfolobales*, *S. islandicus* has a 2–3 Mb circular chromosome with three synchronous origins of replication that are highly regulated to replicate the chromosome once per cell division (Lundgren et al. 2004; Robinson et al. 2004; Robinson and Bell 2007). Comparative genomics studies of seven *S. islandicus* strains from three geographically isolated populations demonstrated that some regions of the single *S. islandicus* chromosome are more variable than others in both gene content and sequence polymorphism, and also that these regions may be associated with distance from the replication origins (Reno et al. 2009; Andersson et al. 2010; Flynn et al. 2010). The evolutionary basis of this nonuniform distribution of variation is unknown, although several mechanisms have been proposed. Here, we demonstrate through comparative genomics that recombination–selection balance results in a mosaic of variation along the genome of *S. islandicus*.

## Materials and Methods

### Identification of Polymorphism and Divergence

Ten strains of *S. islandicus* were isolated from the M.16 acidic hot spring in Kamchatka, Russia, in the year 2000. Genomes were sequenced, assembled, and used to construct a clonal phylogeny previously (Cadillo-Quiroz et al. 2012). The genome sequences were aligned along with *S. solfataricus* P2 using Mauve v.2.3.1 (Darling et al. 2010). Single-nucleotide polymorphisms obtained from core locally collinear blocks (LCBs) in the alignment were used to calculate the number of polymorphisms in 10-kb windows. LCBs that contained a bias of single nucleotide polymorphism (SNP) density toward the edge of the alignment could have been the result of alignment artifacts. Therefore, such blocks were analyzed and SNPs that were contained in regions of poor alignment were discarded (supplementary table S4, Supplementary Material online). The 10-kb window size was chosen to obtain the highest possible resolution of genomic regions without the window size becoming so small that windows with no polymorphisms occurred. Windows that contained alignment gaps totaling greater than 5 kb were not analyzed. Windows of size 5 and 50 kb were analyzed as well and resulted in similar patterns that do not alter conclusions. Estimates of 95% expected intervals are based on binomial distributions using the polymorphism data itself. Any SNPs in which *S. solfataricus* did not share a nucleotide with any of the ten *S. islandicus* strains for that position were assigned as divergent between *S. solfataricus* and *S. islandicus.* Correction for back mutation was performed independently on each window for the non-divergent SNPs using the rate estimated for that window based on divergent SNPs, and this does not alter ranking of windows or Spearman rank correlation analysis. In addition, two windows of exceptionally high polymorphism containing parts of the CRISPR-Cas system and the S-layer gene locus, respectively, were removed to focus on the core, intrapopulation dynamics. The first is an extreme outlier with 31% of sites being polymorphic and 99% being recombinant, likely arising from horizontal gene transfer from outside the population and described in Held et al. (2013). Its omission does not affect conclusions of the study. The S-layer gene is another outlier with high levels of polymorphism and likely horizontal gene transfer and it is described in Cadillo-Quiroz et al. (2012).

### Tests of Selection

dN/dS ratios were calculated for protein-coding genes using SNAP (Korber 2000), comparing M.16.27 and M.16.4 with the outgroup *S. solfataricus*. These two *S. islandicus* represent 70% of the diversity within the population, and using only these two accounts for 99% of the divergence between *S. islandicus* and *S. solfataricus*. The MK test was performed for genes using both fixed and polymorphic sites using the non-synonymous or synonymous substitution determined by direct analysis of individual codons from the whole genome alignment (McDonald and Kreitman 1991). *G*-test values greater than 15.5 were considered statistically significant at *P* <8.2 × 10^−^^5^, based on a Bonferroni correction for the number of genes tested that contained synonymous and non-synonymous polymorphisms (606 genes, *P* < 0.05). Tajima’s *D* was calculated per gene as described in Tajima (1989).

### Inference of Recombination

SNPs were compared with a clonal phylogeny generated in Cadillo-Quiroz et al. (2012). SNPs that did not fit one of the 18 possible configurations indicative of a single mutation on a single branch of the tree were homoplasies, called recombinant SNPs. Although a small subset of recombinant SNPs may result from multiple mutations at the same position, it is estimated that based on the total number of SNPs and the core genome size, multiple mutations can only explain 11 of the 1,669 recombinant SNPs (0.7%) (Maynard Smith and Smith 1998). The biallelic nature of 99.8% of the SNPs confirms the rarity of multiple mutations at a single site. Unique recombinant topologies were calculated in 10-kb windows by counting all the unique polymorphism patterns of the recombinant SNPs in a window.

### Low Recombination Zones

21% of all SNPs in the *S. islandicus* genome are identified as recombinant. Based on this ratio and assuming a null model of a constant rate of recombination across the genome, we estimate that we would observe >30 nonrecombinant SNPs in a row by chance with a probability of <0.001 (0.79^30^). Therefore, we identified LRZs as stretches of the genome that contained >30 consecutive nonrecombinant SNPs. Levels of polymorphism within LRZs were calculated as the number of nonrecombinant polymorphisms contained divided by the core genome length of the LRZ.

### Linkage Disequilibrium and Kelly’s Z_nS_

Linkage disequilibrium was calculated using Kelly’s Z_nS_, described in Kelly (1997). Briefly, linkage disequilibrium is calculated pairwise for all sites in a given set (LRZ), and the mean of these values is the Z_nS_ metric. This value is then compared with a table that yields the significance of these values based on Z_nS_ values resulting from simulated data for the same sample size and number of polymorphisms analyzed. These simulations were generated using MS and Seq-Gen incorporating the population structure between the “red” and “blue” groups described in Cadillo-Quiroz et al. (2012). Linkage disequilibrium analysis of LRZs was performed using DNAsp v.5.10.1 to calculate Kelly’s Z_nS_ (Librado and Rozas 2009). LRZs with linkage disequilibrium were analyzed for the most common polymorphism pattern for the SNPs within the LRZ, and fixed zones were identified as those for which the most common polymorphism was segregated between two sympatric species as described in Cadillo-Quiroz et al. (2012).

### Statistical Analyses

Spearman’s rank correlations and Wilcoxon sign-rank tests were performed using R v.2.13.2 in Rstudio v.0.96.331 using the stats package (Wickham 2009). 95% expected ranges for polymorphism, divergence, and recombination were determined according to the range for a binomial distribution based on the mean levels observed in the polymorphism data. Levels of recombinant polymorphisms were compared with levels of nonrecombinant polymorphisms by a Wilcoxon sign-rank test using paired data for recombinant and nonrecombinant SNPs in all 1-kb windows that contained at least one recombinant SNP. This test determines whether recombination inherently drives the polymorphism level by mechanisms such as mutagenic recombination.

### Simulations

Simulations of sequence data were performed using a combination of MS and Seq-Gen as described in the MS user manual (Rambaut and Grassly 1997; Hudson 2002). Simulations utilized 11 samples, with 1 representing the outgroup using the population structure feature of MS, using the following parameters: -T (tree output for seq-gen), -I 2 1 10 (set population structure), -ej 30 1 2 (populations split to approximate divergence). Mutation rates were used in seq-gen that resulted in values that approximated mean levels of polymorphism and divergence in the natural population via the parameters: -mHKY (default model of substitution), -l (length of window), -s (substitution rate). Substitution rates in the simulations involving variable substitution rates were 0.001, 0.002, and 0.003, while 0.0018 was selected for constant substitution rate simulations.

## Results

### Mosaic of Polymorphism Throughout the *S. islandicus* Genome

Previously, the genomes of 12 *S. islandicus* strains from a single hot spring in the Mutnovsky Volcano region of Kamchatka, Russia, were sequenced. It was found that while there is evidence for recombination between all strains within this population, ten of these comprised two species (red and blue) as defined by higher levels of gene flow within than between the two groups (Cadillo-Quiroz et al. 2012). A whole genome alignment was constructed containing positions shared by these ten highly syntenic *S. islandicus* genomes and the outgroup genome of *S. solfataricus* P2 (She et al. 2001). After partitioning the alignment into 10-kb windows of sequence and removing poorly aligned windows (see Materials and Methods), the data set consists of 216 bins containing 2.0 Mb of core aligned nucleotides, with 8,094 SNPs among 10 *S. islandicus* strains and 201,941 SNPs distinguishing *S. islandicus* from *S. solfataricus* (She et al. 2001).

**Fig. 1.—:**
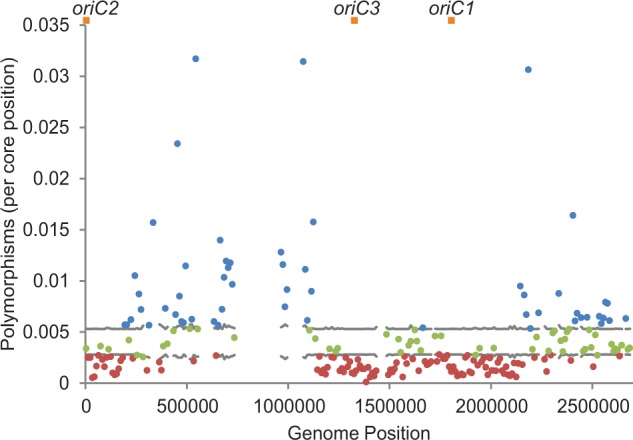
Polymorphisms are unevenly distributed throughout the genome. Polymorphisms in 10-kb windows were plotted along the genome coordinates of M.16.27. Horizontal gray lines indicate the upper and lower bounds of a 95% range of the expected polymorphism level under constant neutral accumulation of polymorphisms. Blue and red dots indicate values above and below this interval, respectively. Points in green fall within the confidence interval of the mean. Orange squares represent the positions of the three origins of replication. Empty windows contain greater than 5 kb of gaps in the core alignment and are therefore not analyzed.

In order to look at the distribution of intrapopulation polymorphisms throughout the genome, levels of polymorphism were compared with a 95% range of values expected around the mean level of polymorphism (fig. 1). Simulation of sequence data under neutral models with constant mutation rate shows that values rarely deviate outside the 95% expected range and are not localized in the genome but are randomly distributed (supplementary fig. S1, Supplementary Material online). In contrast, our genome analysis shows that out of 216 windows, 25% (55) contain high levels of polymorphism and 50% (108) contain low levels of polymorphism. Windows with high levels of polymorphism fall within the two previously described regions of variable gene content (Reno et al. 2009), while windows with low polymorphism are distributed throughout the genome, focused especially in regions that include the three origins of replication. Similar patterns were observed within the red and blue *S. islandicus* species when analyzed independently (supplementary fig. S2, Supplementary Material online).

The observed variation in polymorphism across the genome could result from the interplay between three evolutionary mechanisms: 1) variation in the rates at which mutations are introduced, 2) variation in selection, that is, the level of functional constraint on proteins in different genomic regions, and 3) variation in rates of recombination across the chromosome either by introducing new variation or by unlinking polymorphisms. We tested each of these hypotheses in turn by investigating their unique genomic signatures (Webster and Hurst 2012).

### Variation in Mutation Rates is Not the Cause for the Mosaic of Polymorphism

**Fig. 2.—:**
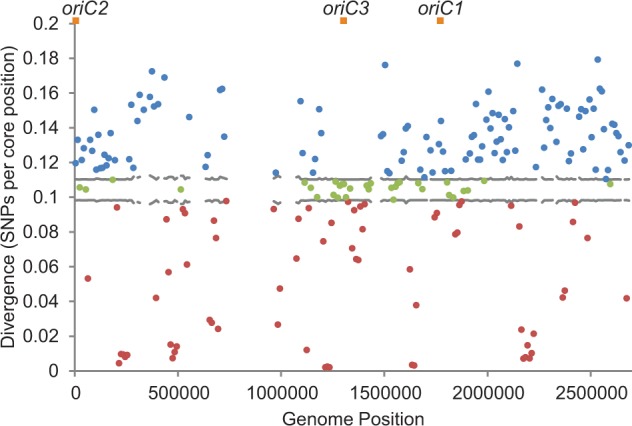
Divergence between *Sulfolobus islandicus* and *S. solfataricus*. Divergence was calculated for 10-kb windows as counts of nucleotides in *S. solfataricus* with no match in *S. islandicus*. Horizontal gray lines indicate the 95% range of the expected divergence level if the mutation rate were constant throughout the genome. Blue and red dots indicate values above and below this interval, respectively. Points in green fall within the confidence interval of the mean. Orange squares indicate origins of replication. Empty windows contain greater than 5 kb of gaps in the core alignment and are therefore not analyzed.

If variation in mutation along the chromosome were the cause of the observed mosaic, we would expect to see a positive correlation between within-population polymorphism and divergence from an outgroup species (Roselius et al. 2005). Simulations of sequence data using multiple mutation rates varying by genomic location support this expectation, showing a strongly significant positive correlation between divergence and polymorphism (*P* < 2.2 × 10^−^^16^, rho = 0.54, supplementary fig. S3, Supplementary Material online). We compared patterns of polymorphism among our ten *S. islandicus* strains with patterns of divergence between these strains and the genome of *S. solfataricus* (She et al. 2001). From 216 windows, 54% (116) had higher divergence and 31% (68) had lower divergence than the 95% range of values expected if the mutation rate was constant (fig. 2). However, windows with low and high polymorphism did not coincide with windows of low and high divergence, and we found no correlation between levels of polymorphism and divergence per window (Spearman rank correlation: *P* = 0.95, rho = −0.11). These data show that variation in polymorphism across the genome is not explained by variation in mutation rates around the chromosome.

### Variation in Selection Influences Levels of Polymorphism on a Regional Scale

If the pattern of polymorphism is a reflection of variation in selection across the genome, we predict that purifying selection would be stronger in regions of low variation, while diversifying selection or relaxed purifying selection would allow for increased polymorphisms in other regions. We note that if functional constraint were consistent between *S. solfataricus* and *S. islandicus* and variation in selection were the cause of variation in polymorphism, we would expect to see a correlation between polymorphism and divergence above. As divergence and polymorphism are not correlated, it is unlikely that variation in selection is the sole driving force in defining the observed mosaic of polymorphism.

**Fig. 3.—:**
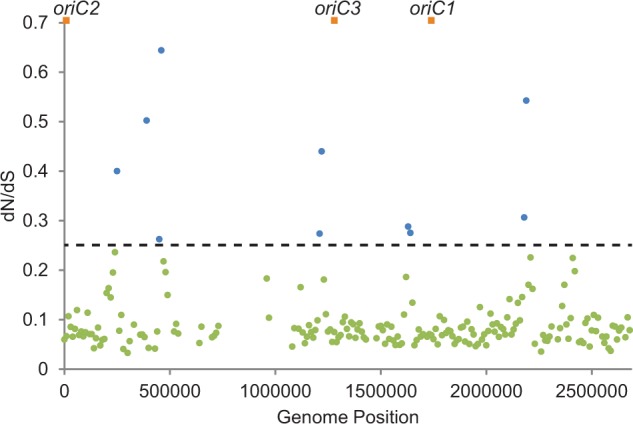
Variation in genome-wide selection. Mean pairwise dN/dS ratios shown for protein-coding genes in 10-kb windows between *Sulfolobus islandicus* and *S. solfataricus*. Horizontal dotted line indicates the upper bound of the 95% confidence interval of the mean for a normal distribution of dN/dS values. Orange squares indicate origins of replication. Empty windows either lack sufficient core sites for analysis or genes containing variation.

Overall, we find little evidence for diversifying selection between the red and blue species of *S. islandicus* (Cadillo-Quiroz et al. 2012)*.* Per-gene analyses with Tajima’s *D* and the McDonald–Kreitman test find few possible candidates for diversifying selection in these genomes, which cannot explain genome-wide patterns in the high polymorphism regions. We therefore examined evidence of variation in the strength of purifying selection across the genome using dN/dS. We predict that a positive correlation between the dN/dS ratio and polymorphism in genomic windows would indicate that genes in certain regions of the genome are under stronger purifying selection than others. Signatures of selection among genomes within the population show no significant correlation between dN/dS and polymorphism levels (supplementary table S1, Supplementary Material online), which could be due to limitations of using dN/dS over such short time scales (Rocha et al. 2006; Kryazhimskiy and Plotkin 2008).Therefore, we expanded dN/dS comparisons to those between *S. islandicus* and *S. solfataricus* to provide higher resolution and evidence of selection over longer time scales. In this comparison, we do find a significant correlation between dN/dS values and levels of polymorphism in 10-kb windows (Spearman rank correlation: *P* = 1.4 × 10^−^^3^, rho = 0.21, fig. 3). However, this correlation is highly dependent on the current localization and linkage of genes, as the correlation is greatly reduced when genes are randomly permuted between windows (supplementary table S2, Supplementary Material online). Consistent with this regional pattern, there is also a similar correlation between dN/dS values and levels of intergenic polymorphism (*P* = 1.6 × 10^−^^3^, rho = 0.20), supporting the observation that varying purifying selection may be influencing polymorphism on a regional scale within the genome. This connection between selection and regional levels of polymorphism implies that linkage is a factor in how selection influences these genomes.

### Variation in Recombination Correlates with the Mosaic of Polymorphism

**Fig. 4.—:**
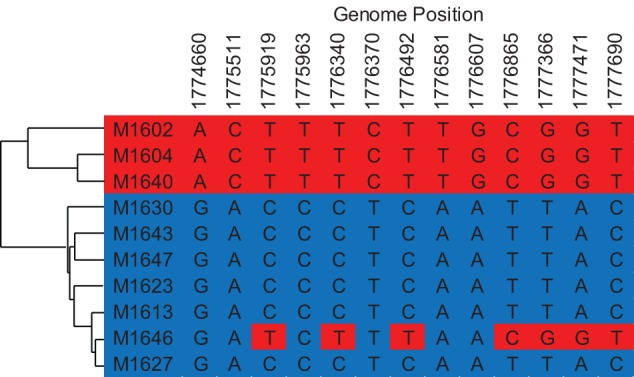
Identification of recombinant SNPs. A small illustrative sample of SNP data from the genome alignment. Genome positions of SNPs in M.16.27 are listed at the top. The phylogenetic tree on the left corresponds to the topology of the clonal genealogy constructed using ClonalFrame data (Cadillo-Quiroz et al. 2012). Recombination events between the “red” strains and M.16.46 are apparent in the data, as is the discontinuity of the relationship over the length of the sequence.

The interaction of recombination and selection results in mosaics of polymorphism when recombination rates vary across the chromosome (Felsenstein 1974). Higher levels of recombination allow for the efficient action of selection thereby targeting reductions of genetic polymorphism to the selected locus, whereas low levels of recombination subject neighboring polymorphisms to the effects of background selection or genetic hitchhiking. To explore variation in the amount of recombination around the chromosome, 1,669 recombinant SNPs (homoplasies) were identified by having a conflicting phylogenetic topology to the core nucleotide tree (fig. 4). This nonparametric method of investigating recombination measures recombination among the ten sequenced members of the population, and it is useful for analyzing closely related taxa that have had chances to exchange genes in their evolutionary history. Recombination events appear to occur in a discontinuous fashion with short tracts (fig. 4). This observation of multiple discontinuous short tracts interspersed with recipient genome sequence is consistent with previous studies of recombination through experimental transformation in *Sulfolobus* (Grogan and Rockwood 2010). Recombinant SNPs also do not show increased frequencies of polymorphisms within windows above local nonrecombinant polymorphism levels, indicating that they are not inherently mutagenic or driving the polymorphism levels intrinsically (Wilcoxon sign-rank test, *P* = 0.92, see Materials and Methods).

**Fig. 5.—:**
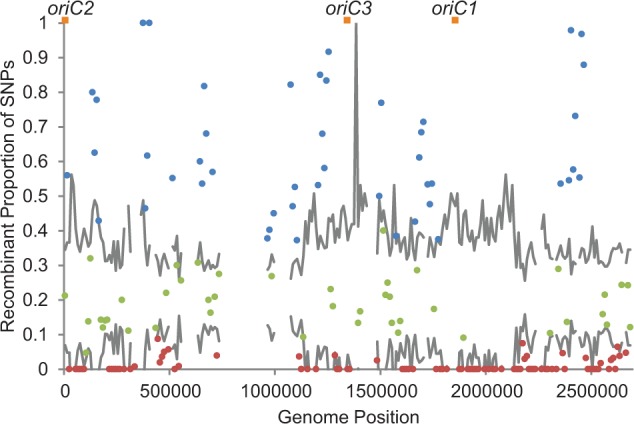
Recombination varies throughout the *Sulfolobus islandicus* genome. The ratio of recombinant SNPs to total SNPs in 10 kb windows was plotted along the genome coordinates of M.16.27. Gray horizontal lines indicate the 95% expected range of the recombinant SNP proportion based on constant recombination rate and varying number of present polymorphisms depending on the window. The interval varies widely due to its dependence on the variable number of polymorphisms within each window. Blue dots and red dots indicate values above and below the 95% expected range, respectively. Green points fall within the confidence interval of the mean. Windows with no recombinant SNPs that fall within the interval are not shown for clarity. Orange squares indicate origins of replication. Windows with greater than 5 kb of gaps in the alignment were not analyzed and are left empty.

For each 10-kb window, the proportion of recombination was computed as the ratio of the number of recombinant SNPs to the total number of SNPs in order to normalize the influence of the local level of polymorphism on detection of recombination. As shown in figure 5, recombination is evident throughout much of the genome; however, the proportion of recombination varies considerably between regions, with regions of low recombination occurring in regions of low polymorphism. The normalized number of recombinant SNPs shows a significant, positive correlation with levels of polymorphism (Spearman’s rank correlation: *P* = 5.5 × 10^−^^6^, rho = 0.29). This distribution of recombination around the genome is supported by levels of recombination within the red and blue species individually, although there are far fewer such SNPs within each individual group (Spearman rank correlation: red *P* = 9.8 × 10^−^^13^, rho = 0.47; blue *P* = 1.1 × 10^−^^7^, rho = 0.39, supplementary fig. S4, Supplementary Material online). An alternative measure of recombination that functions independently of polymorphism levels is the number of distinct phylogenetic incongruences from the core gene tree observed per window (also normalized by number of SNPs; supplementary fig. S5, Supplementary Material online). This distribution also correlates significantly with the polymorphism levels (Spearman rank correlation: *P* = 3.8 × 10^−^^8^, rho = 0.36).

**Fig. 6.—:**
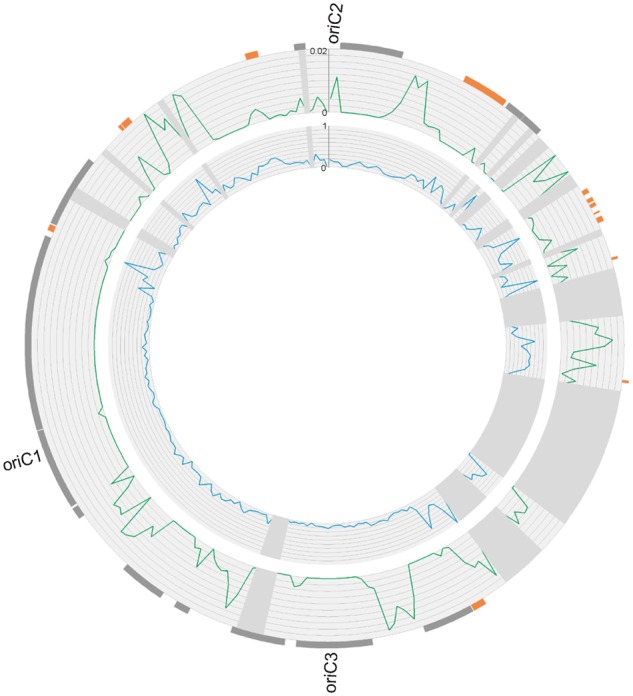
Low recombination zones throughout the genome. LRZs were plotted as tiles on the genome coordinates of M.16.27 on the outermost ring. Orange tiles correspond to LRZs with elevated levels of polymorphism, and gray tiles correspond to LRZs with low levels of polymorphism. The next ring shows levels of recombination (green) as determined by the number of recombinant SNPs divided by total polymorphic sites in 10-kb windows. The next ring shows levels of polymorphism (blue) as number of polymorphic sites per total core nucleotide positions in 10-kb windows. Gray wedges indicate windows with greater than 5 kb of gaps in the alignment and were not analyzed. Figure generated using Circos (Krzywinski et al. 2009).

There are large zones of the genome where no recombinant SNPs are present (fig. 6). We quantitatively defined low recombination zones (LRZs) as statistically significant stretches of sequence that lack recombinant SNPs. In total, 35 LRZs were identified ranging in size from 2,738 to 281,301 bp with a median of 17,343 bp. All three origins of replication fall within LRZs, with *oriC3* and *oriC1* in low polymorphism zones of size 109,799 and 117,463 bp, respectively, and *oriC2* located in a smaller average polymorphism zone of size 8,894 bp. The three largest LRZs in the genome either contain or fall within 50 kb of an origin of replication (fig. 6). The majority of the remaining LRZs contain lower levels of polymorphism than the rest of the genome, and there is a strong negative correlation between levels of polymorphism and LRZ size (Spearman rank correlation, *P* = 9.9 × 10^−^^7^, rho = −0.73). These data are consistent with a model of background selection, because larger LRZs have more potential for deleterious mutations to remove linked polymorphism from the population.

Not all of the LRZs contain low polymorphism; 13 have polymorphism levels that are significantly higher than the rest of the genome. All but one of these are located near highly recombining regions of the genome. Interestingly, six of these high polymorphism LRZs show significant levels of linkage disequilibrium (supplementary table S3, Supplementary Material online). This suggests that these LRZs may result from the recent and rare import of a divergent allele from outside of the population included in this study, which would result in the introduction of polymorphism but not detectable recombinant SNPs (Didelot et al. 2010; Flynn et al. 2010). An analysis of all high polymorphism windows detects significant evidence of linkage disequilibrium in 36% of windows. This suggests that higher recombination in these regions may introduce variation as well as preserve it through recombination–selection balance.

## Discussion

Recombination–selection balance describes the degree to which selection on one position removes linked neutral polymorphisms balanced against the action of recombination to unlink neutral polymorphism and preserve diversity. Regions of low recombination are expected to sustain drops in polymorphism due to background selection or genetic hitchhiking on large linked regions of the genome, although these two types of selection are difficult to distinguish (Andolfatto 2001; Innan and Stephan 2003). Studies on sexual eukaryotes, especially in *Drosophila* spp., have shown a correlation between recombination rates and genetic diversity throughout the genome (Begun and Aquadro 1992; McGaugh et al. 2012), causing a difference in polymorphism approximately one order of magnitude.

Similarly, we show that the influence of recombination varies across the chromosome in *Sulfolobus islandicus.* This variation in recombination is correlated with variation in levels of polymorphism, and on a similar scale as that seen in *Drosophila*. Reduced polymorphism in regions of low recombination combined with a regional correlation between dN/dS values and both genic and intergenic polymorphism suggests that purifying selection is removing variation through background selection. The discontinuous pattern of recombination (fig. 4) found throughout the genome and demonstrated by others experimentally (Grogan and Rockwood 2010) suggests that these regions of low recombination are not due to long recombination tracts as observed in other systems (Naor et al. 2012; DeMaere et al. 2013) but rather chromosome architecture decreases recombination on a regional scale, particularly around the three origins of replication. It is not clear why there is decreased recombination around the origins of replication in *S. islandicus*. Andersson et al. (2010) propose an intriguing hypothesis that recombination-based repair between newly replicated, identical sister chromosomes at the origins of replication results in decreased levels of polymorphism. This is based on observed hemicatenanes that occur at origin regions (Robinson et al. 2007) and the fact that *Sulfolobus* cells spend approximately 65% of their cell cycle in this extended G2-phase, where the newly replicated chromosomes are not yet segregated (Bernander 2007). This recombinational repair-based mutational bias predicts a positive linear correlation between polymorphism and divergence under neutrality, which is not supported by our data. However, if interacting newly replicated sister chromosomes excluded recombination with foreign DNA, this could explain why recombination observed in this study is reduced around the three replication origins in *Sulfolobus*.

Previous studies of *S. islandicus* have identified a mosaic of polymorphism across the genomes of more divergent and non-sympatric sequenced strains (Andersson et al. 2010; Flynn et al. 2010). These studies suggested several hypotheses based on mutational biases or selective constraints. We show here that mutational biases are unlikely to explain the observed patterns via the comparisons between *S. islandicus* and *S. solfataricus*. We did find that selection plays a role in the relative levels of diversity among regions, but its effect acts through recombination to create regional patterns rather than solely on individual genes themselves.

In addition, it was suggested that, in regions distal to the origins, variation may be introduced by recombination with divergent alleles (Flynn et al. 2010). We did identify two examples where we observe the import of highly divergent alleles at both the CRISPR loci (Held et al. 2013) and the S-layer locus (Cadillo-Quiroz et al. 2012) in some members of this population. However, these two outliers are the only ones in which variation is greater than 10-fold the average across the genome. Our finding of some evidence of linkage disequilibrium in highly polymorphic regions suggests that variation may also be introduced into the genome from divergent taxa outside of our population. This appears to be localized in highly recombining regions of the genome, providing another mechanism through which recombination influences the architecture of divergence in these organisms. These regions may be particularly interesting in understanding divergence between the two species of *S. islandicus* found in this hot spring, as half of these divergent alleles are fixed between the species (Cadillo-Quiroz et al. 2012).

In *S. islandicus*, as in other archaea, core genes involved in essential housekeeping functions are co-localized near the origins of replication that we have shown to have low polymorphism and low recombination (Andersson et al. 2010; Flynn et al. 2010; Pelve et al. 2012), while transiently useful, strongly selected genes such as those in immunity and viral resistance are co-localized in more variable regions with higher recombination (Reno et al. 2009; Held et al. 2013). The data reported here indicate that the evolutionary mechanism that is shaping this genome architecture may be variation in recombination. As it is advantageous to have genes under strong selection in regions of high recombination where selection is more efficient, this genome organization may be selected for over time.

This study presents a population-level analysis of the genomic architecture of divergence in archaea. Although *Sulfolobus*, like bacteria and other archaea, do not reproduce sexually, recombination is frequent enough to create a discontinuous signature of interacting evolutionary forces in the genome. Future work integrating well-known molecular mechanisms of recombination, mutation, and replication with patterns of natural variation driven by selection, migration, and genetic drift will provide insights into the factors that define the topology of divergence and the genetic basis of adaptation and speciation in asexual but recombining microorganisms.

## Supplementary Material

Supplementary figures S1–S5 and tables S1–S4 are available at *Genome Biology and Evolution* online (http://www.gbe.oxfordjournals.org/).

Supplementary Data
