# Supplementary material for: Recombination Shapes Genome Architecture in an Organism from the Archaeal Domain
Source: Genome Biol Evol. 2014 Jan 3;6(1):170–8. doi: 10.1093/gbe/evu003 (PMC3914695; doi:10.1093/gbe/evu003)
Supplement: Supplementary Data [file supp_6_1_170__index.html]

Recombination Shapes Genome Architecture in an Organism from the Archaeal Domain — Supplementary Data 

# Recombination Shapes Genome Architecture in an Organism from the Archaeal Domain

## Supplementary Data

files

**Files in this Data Supplement:**

- Supplementary Data - pdf file
- Supplementary Data - pdf file
