# Supplementary material for: Recombination Shapes Genome Architecture in an Organism from the Archaeal Domain
Source: Genome Biol Evol. 2014 Jan 3;6(1):170–8. doi: 10.1093/gbe/evu003 (PMC3914695; doi:10.1093/gbe/evu003)
Supplement: Supplementary Data [file supp_evu003_Supplementary_Figures_GBE.pdf]

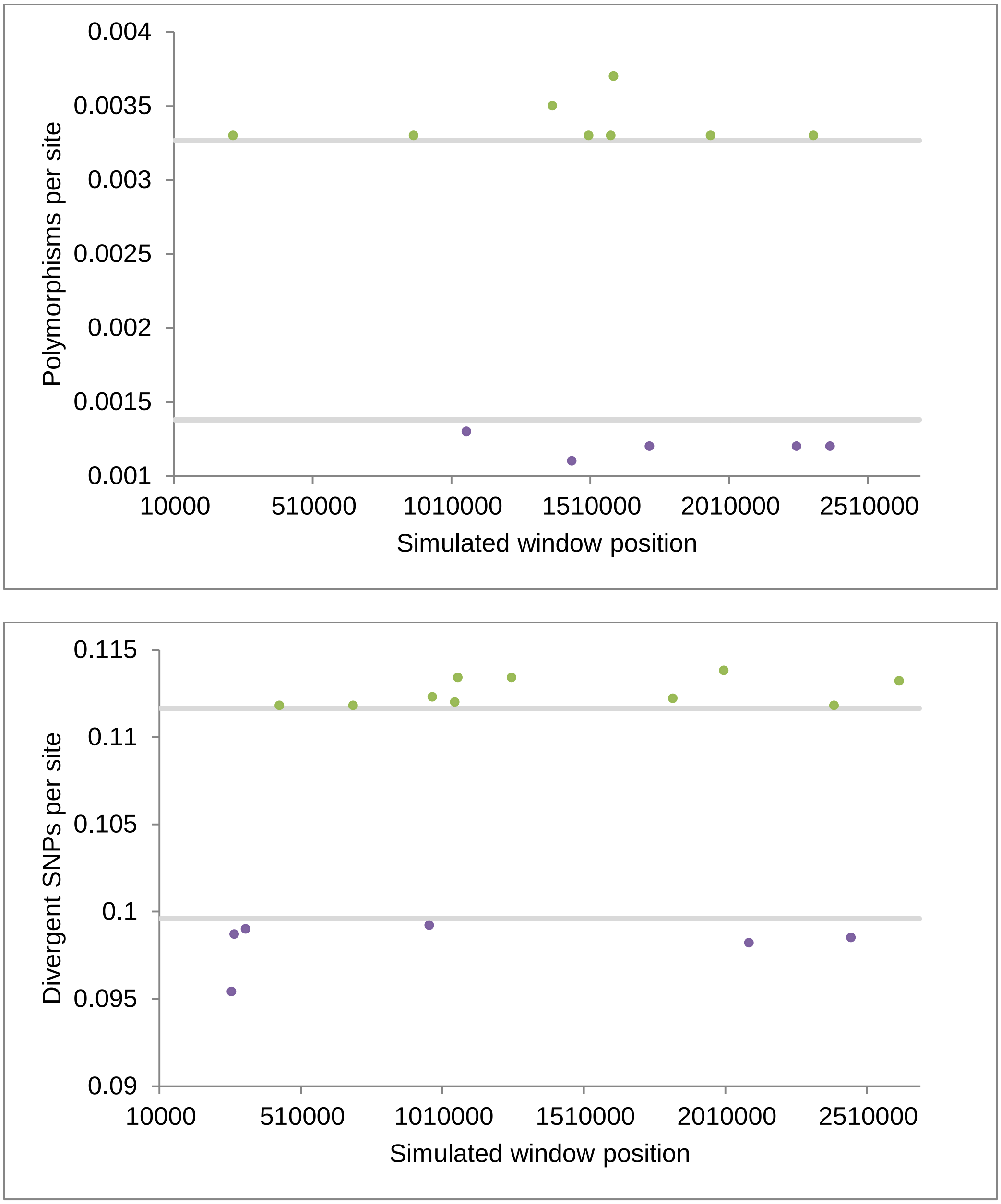

Figure S1 – Simulations with constant substitution rate. A) Polymorphisms per site calculated in 10Kb windows over a 2,692,402 bp simulation of constant substitution rate. B) Divergence between the subpopulation and outgroup. Grey bars indicate 95% expected range of values according to a binomial distribution.

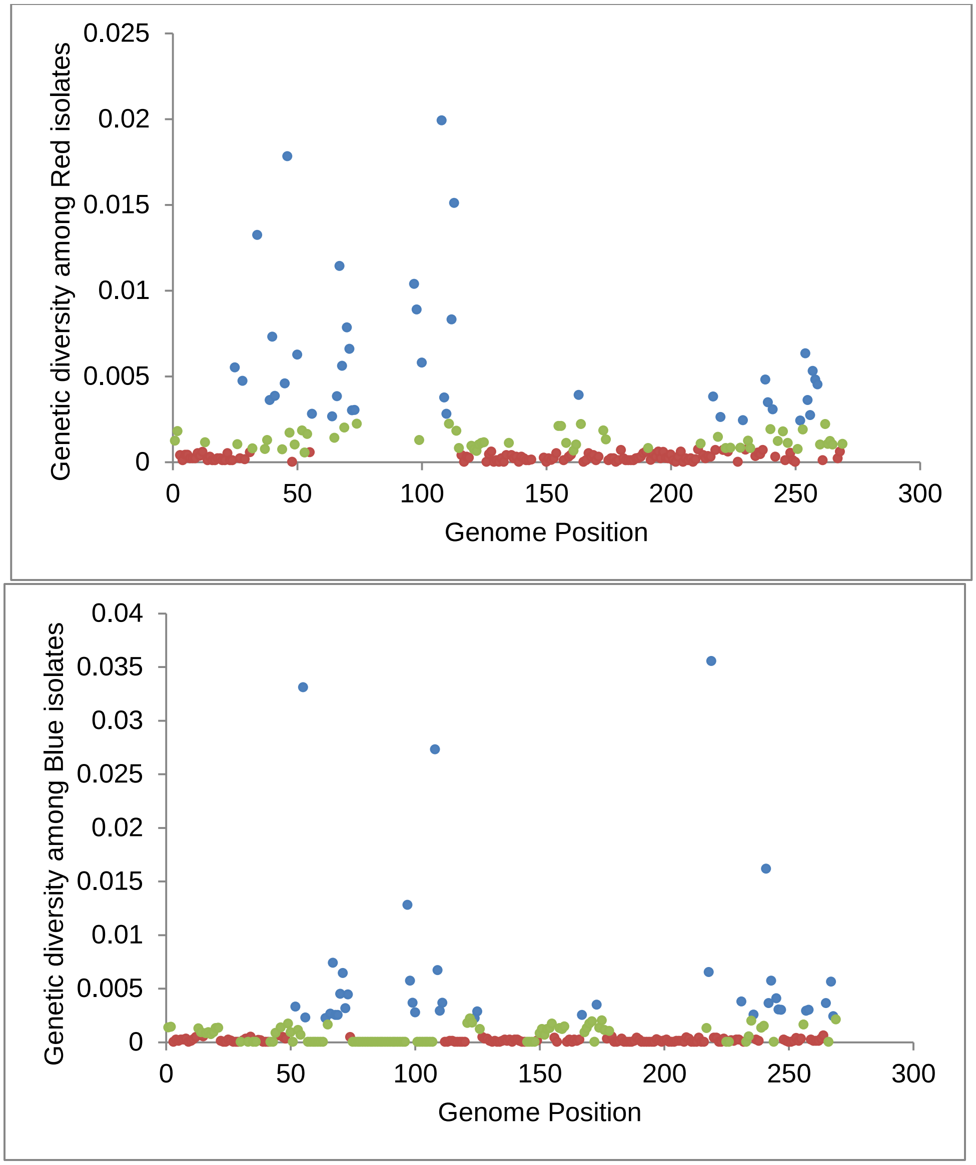

Figure S2 – Levels of polymorphism within the two coexisting species show similar patterns to each other, and with the patterns of polymorphism among all ten genomes. Genome position is the number of the 10Kb window analyzed. Comparison between the within-red and within-blue datasets shows a significant positive correlation (Pearson correlation: $p=9.7x10^{-7}$, R=0.32). Both also show a correlation back to the full population polymorphism distribution (Pearson correlation: red vs. total $p<2.2x10^{-16}$, R=0.69; blue vs. total $p<2.2x10^{-16}$, R=0.79).

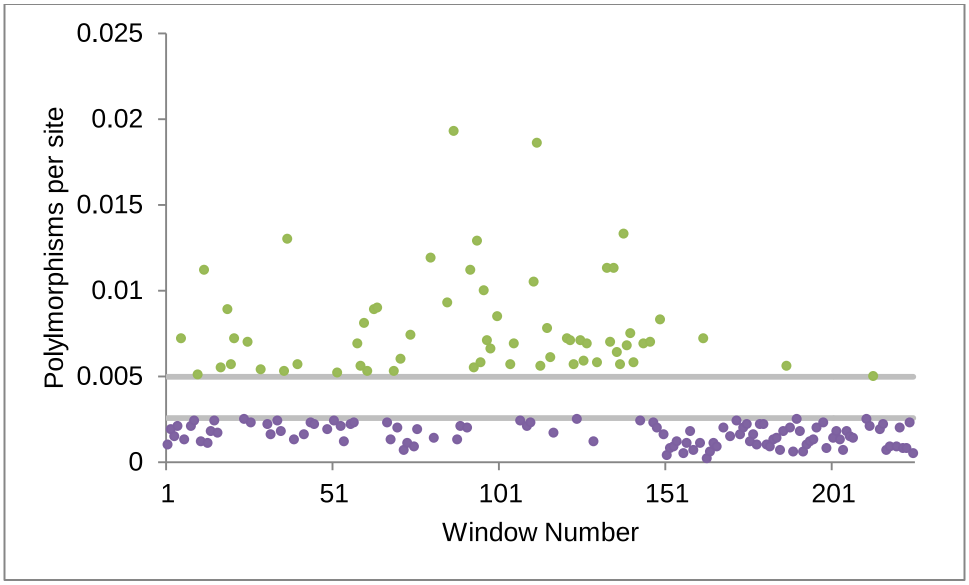

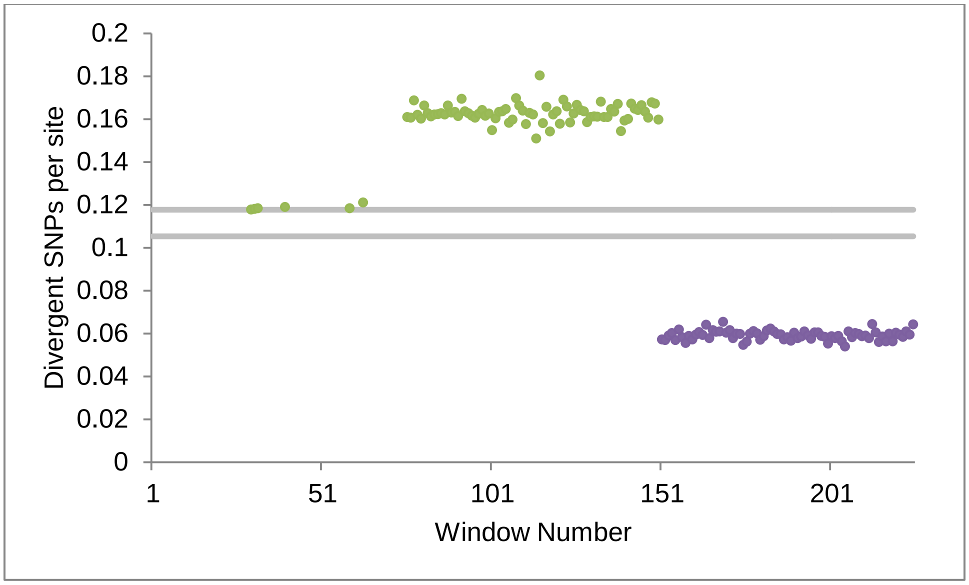

Figure S3 – Simulated data under multiple substitution rates. A) Polymorphism calculated in each simulated 10Kb window. B) Divergence calculated in each simulated 10Kb window. Horizontal grey lines indicate the 95% expected range of values according to a binomial distribution. Green and purple dots indicate points above and below the expected range, respectively. Window numbers between the two plot correspond to one another and are drawn from the same simulation. The parameters used to generate the simulated population are described in the methods.

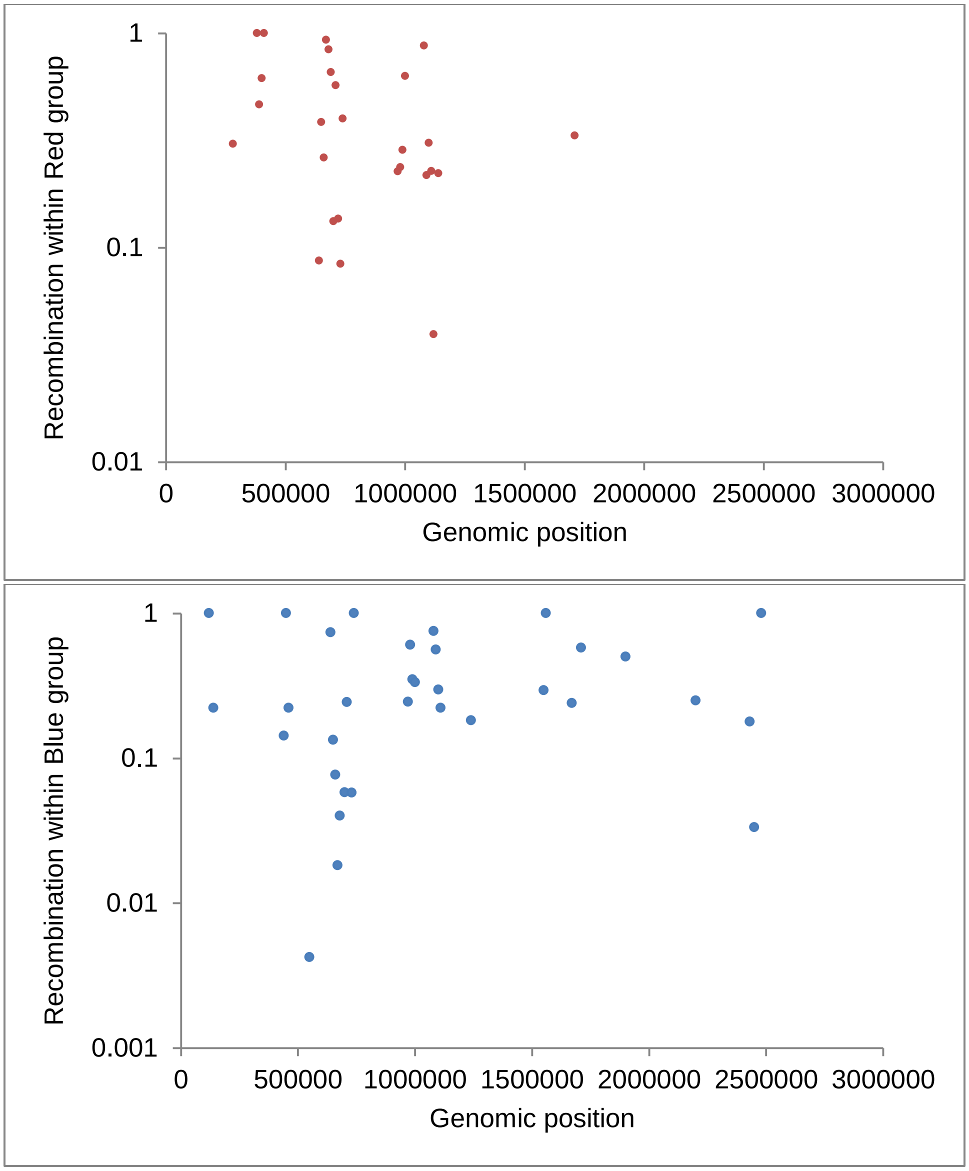

Figure S4 – Recombination estimated within subpopulations. There are limited events by this method of detection for recombination within subpopulations, leading to limited resolution of within-subpopulation recombination patterns. SNPs which violate the core gene tree topology within a subpopulation, but are not explained by singleton mutations, are mapped here, as recombinant SNPs per total within-group SNPs in 10Kb windows. No value indicates either no recombination data for that window.

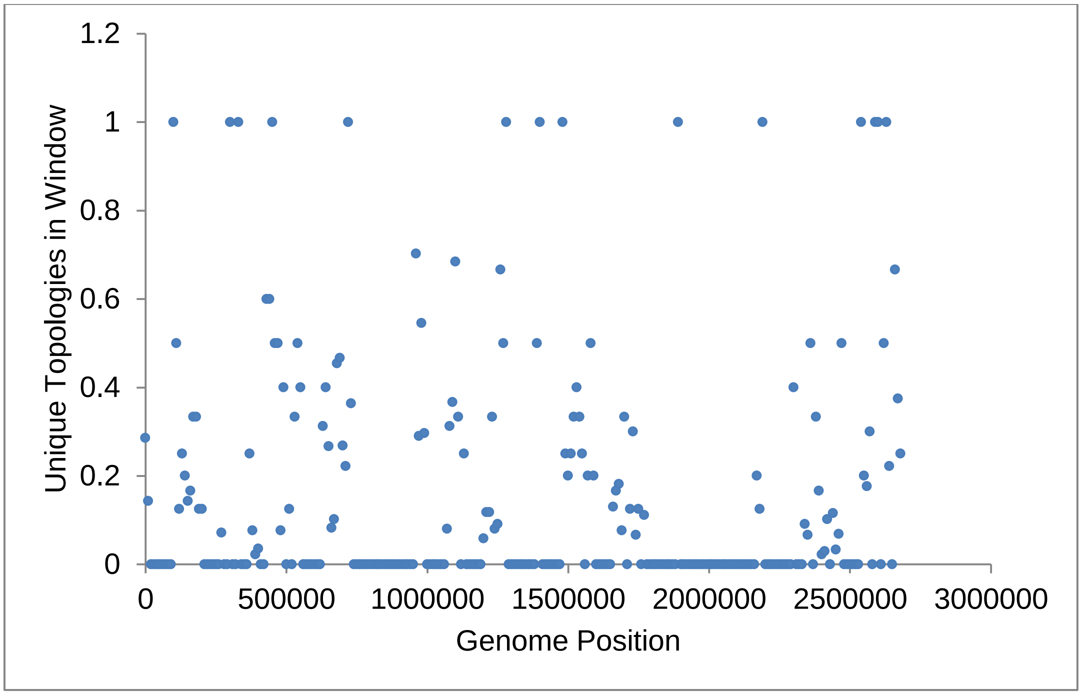

Figure S5 – Recombination estimated using the number of unique recombinant topologies in each 10Kb window. Recombinant SNPs in each 10Kb window were analyzed for unique phylogenetic topologies, and this number was selected to estimate the number of recombination events that have occurred in each window. Windows with less than 5Kb of core nucleotide positions are empty. Values were corrected for the number of total recombinant SNPs in each window.
