# Supplementary material for: Recombination Shapes Genome Architecture in an Organism from the Archaeal Domain
Source: Genome Biol Evol. 2014 Jan 3;6(1):170–8. doi: 10.1093/gbe/evu003 (PMC3914695; doi:10.1093/gbe/evu003)
Supplement: Supplementary Data [file supp_evu003_Supplementary_Tables_GBE_revision2.pdf]

Table S1 – Selection over short time scales shows no correlation with polymorphism levels

Test of selection	Polymorphism level	p-value	rho
dN/dS	Within-Blue	0.42	-0.014
dN/dS	Within-Red	0.60	0.017
Tajima's D	Whole Population	0.96	-0.12

dN/dS calculated as mean pairwise dN/dS ratio for genes within 10Kb windows between the Red and Blue species of *S. islandicus*. Within-Blue and Within-Red polymorphisms distinguish strains within the given species. Whole population polymorphisms are polymorphic sites between any of the ten genomes. p-value and rho are the result of Spearman rank correlation analyses between the value given from the test of selection and the particular polymorphism level over all 10Kb windows.

Table S2 – Random permutations of gene locations influence regional correlations between dN/dS and polymorphism

Trial No.	dN/dS range	Polym range	dN/dS stdev	Polym stdev	p-value	rho
Actual	*0.61*	*0.030*	*0.082*	*0.0028*	$1.2x10^{-3}$	*0.21*
1	0.62	0.051	0.064	0.0037	0.17	0.066
2	0.62	0.027	0.060	0.0026	$6.7x10^{-3}$	0.17
3	0.60	0.025	0.065	0.0022	$7.2x10^{-4}$	0.22
4	0.61	0.078	0.062	0.0055	$7.8x10^{-3}$	0.17
5	0.59	0.031	0.063	0.0025	0.080	0.097
6	0.57	0.031	0.060	0.0026	0.052	0.11
7	0.57	0.026	0.061	0.0023	0.11	0.085
8	0.65	0.055	0.066	0.0048	0.016	0.15
9	0.57	0.12	0.057	0.0084	0.13	0.080
10	0.66	0.011	0.065	0.0016	0.092	0.093
11	0.63	0.058	0.059	0.0042	0.62	-0.022
12	0.64	0.024	0.060	0.0021	$8.3x10^{-3}$	0.17
13	0.57	0.029	0.084	0.0025	0.066	0.11
14	0.57	0.021	0.067	0.0020	$8.3x10^{-3}$	0.17
15	0.59	0.017	0.061	0.0018	0.030	0.13

Random permutations were performed for all genes with contained polymorphism and dN/dS values kept paired. Top row indicates the actual values from the genomic data. Only one random permutation shows a stronger correlation between dN/dS and polymorphism, and most are no longer significant at $p<0.01$.

Table S3 – LRZs with high polymorphism levels and significant linkage disequilibrium

Left end	Right end	Length	# of SNPs	ZnS	Most common topology	Genomic location
461,792	467,365	5,573	43	0.69	Fixed	High polym.
475,153	477,891	2,738	32	0.84	Fixed	High polym.
544,515	548,407	3,892	201	0.86	M.16.43	High polym.
1,110,587	1,130,248	19,661	228	0.68	M.16.4 & M.16.40	High polym.
2,186,632	2,195,873	9,241	256	0.96	Blue (except M.16.30)	High polym.
2,364,760	2,369,684	4,924	30	0.69	Fixed	High polym.

Polymorphism is calculated as SNPs divided by LRZ length. Z_{nS} is a measure of linkage disequilibrium, considered significant at $Z_{nS}>0.67$. Most common topology describes which strains likely acquired the polymorphisms; fixed is between species according to (Cadillo-Quiroz et al. 2012). All of these are found in high polymorphism regions of the genome.

Table S4 – SNPs discarded due to poor alignment quality

LCB Start	LCB Stop	Length	p-value	# removed SNPs
549,279	550,289	1,010	$2.3x10^{-32}$	25
666,967	692,121	25,154	$4.2x10^{-5}$	94
726,208	726,829	621	$3.5x10^{-5}$	15
727,104	728,389	1,285	$1.9x10^{-8}$	12
960,920	962,067	1,147	$3.5x10^{-7}$	3
2,189,205	2,197,273	8,068	$2.6x10^{-26}$	58
2,235,391	2,241,194	5,803	$3.5x10^{-6}$	13
2,631,195	2,653,626	22,431	$7.4x10^{-40}$	146

p-value corresponds to a one-sample t-test comparing the distance from the LCB edge of all SNPs within an LCB to the expected mean (1/4 of the LCB length). After a Bonferroni correction for 294 LCBs containing greater than one polymorphism, the significance cutoff is at $p<1.7x10^{-4}$.
